# Nucleoplasmic lamins define growth-regulating functions of lamina-associated polypeptide 2α in progeria cells

**DOI:** 10.1242/jcs.208462

**Published:** 2018-02-01

**Authors:** Sandra Vidak, Konstantina Georgiou, Petra Fichtinger, Nana Naetar, Thomas Dechat, Roland Foisner

**Affiliations:** Max F. Perutz Laboratories (MFPL), Center of Medical Biochemistry, Medical University of Vienna, Vienna Biocenter (VBC), Dr. Bohr-Gasse 9, A-1030 Vienna, Austria

**Keywords:** Lamin A, Progeria, Premature aging, LAP2α, Nucleoplasmic lamins, Cell proliferation

## Abstract

A-type lamins are components of the peripheral nuclear lamina but also localize in the nuclear interior in a complex with lamina-associated polypeptide (LAP) 2α. Loss of LAP2α and nucleoplasmic lamins in wild-type cells increases cell proliferation, but in cells expressing progerin (a mutant lamin A that causes Hutchinson–Gilford progeria syndrome), low LAP2α levels result in proliferation defects. Here, the aim was to understand the molecular mechanism governing how relative levels of LAP2α, progerin and nucleoplasmic lamins affect cell proliferation. Cells from progeria patients and inducible progerin-expressing cells expressing low levels of progerin proliferate faster than wild-type or lamin A-expressing control cells, and ectopic expression of LAP2α impairs proliferation. In contrast, cells expressing high levels of progerin and lacking lamins in the nuclear interior proliferate more slowly, and ectopic LAP2α expression enhances proliferation. However, simultaneous expression of LAP2α and wild-type lamin A or an assembly-deficient lamin A mutant restored the nucleoplasmic lamin A pool in these cells and abolished the growth-promoting effect of LAP2α. Our data show that LAP2α promotes or inhibits proliferation of progeria cells depending on the level of A-type lamins in the nuclear interior.

This article has an associated First Person interview with the first author of the paper.

## Abstract (precis)

**Highlighted Article:** Lamina-associated polypeptide (LAP) 2α promotes or inhibits proliferation of progeria cells depending on the absence or presence of lamin A in the nuclear interior.

## INTRODUCTION

The nuclear lamina in metazoan cells is a proteinaceous meshwork underlying the inner nuclear membrane, which defines the mechanical properties of the nucleus (Osmanagic-Myers et al., 2015) and regulates chromatin organization and gene expression (Gruenbaum and Foisner, 2015; Kind et al., 2015). Lamins and various lamin-binding proteins of the inner nuclear membrane are the major components of the lamina. Based on their biochemical properties, sequence similarities and expression patterns, lamins are classified into A-type and B-type lamins (Gruenbaum and Foisner, 2015). In mammals, B-type lamins (lamin B1 and lamin B2) are encoded by different genes (*LMNB1* and *LMNB2*, respectively) and are expressed in most embryonic and adult cells. A-type lamins are encoded by *LMNA*, which gives rise to two major isoforms, lamin A and lamin C, primarily expressed at later stages of development and in differentiated cells (Gruenbaum and Foisner, 2015). B-type lamins and lamin A are expressed as prelamins and undergo multiple steps of post-translational processing at their C-terminal CaaX sequence. The first steps are common to B-type lamins and lamin A and include farnesylation of the cysteine residue by farnesyltransferase, followed by cleavage of the aaX tripeptide by FACE1 (also known as ZMPSTE24) or FACE2 (RCE1) and carboxymethylation of the cysteine by isoprenyl-cysteine-carboxymethyltransferase (Rusinol and Sinensky, 2006). B-type lamin processing ends at this step, resulting in farnesylated and carboxymethylated mature B-type lamins, which are tightly associated with the inner nuclear membrane. In contrast, prelamin A is further processed by FACE1, removing 15 C-terminal residues including the farnesylated and carboxymethylated cysteine (Pendás et al., 2002). Therefore, mature lamin A and lamin C, which lacks a CaaX box, are not farnesylated and, in addition to their localization at the lamina, are also found in the nuclear interior (Dechat et al., 2010; Kolb et al., 2011; Moir et al., 2000; Naetar et al., 2017).

The many functions of lamins in nuclear mechanics, chromatin organization and gene expression can, at least in part, be explained by their interaction with numerous lamin-binding proteins (Wilson and Foisner, 2010). Although most of the identified lamin-binding proteins are components of the inner nuclear membrane (Korfali et al., 2012), lamina-associated polypeptide (LAP) 2α, a non-membrane bound isoform of the LAP2 family, interacts with lamin A/C in the nuclear interior (Dechat et al., 2004, 1998, 2000). The presence of LAP2α is essential for maintaining the nucleoplasmic pool of A-type lamins in interphase nuclei (Naetar et al., 2008). LAP2α and A-type lamins in the nuclear interior interact with chromatin (Dechat et al., 1998; Gesson et al., 2016; Vlcek et al., 1999) and with retinoblastoma protein (pRb), an important cell cycle regulator (Dorner et al., 2006; Markiewicz et al., 2002; Naetar and Foisner, 2009). The interaction of pRb with A-type lamins and LAP2α regulates its localization and stability (Markiewicz et al., 2002) and affects pRb-dependent repression of E2F/pRb target genes (Dorner et al., 2006). Thus, nucleoplasmic lamin A/C–LAP2α complexes are thought to function in cell cycle regulation (Gesson et al., 2014). In line with this model, knockout of *Lap2α* (also known as *Tmpo*) in mice leads to hyperproliferation of tissue progenitor cells *in vivo* and to impaired cell cycle arrest in culture (Naetar et al., 2008; Pekovic et al., 2007), whereas LAP2α overexpression decreases cell proliferation (Dorner et al., 2006). Interestingly, in post-mitotic senescent or differentiated cells, LAP2α expression is reduced and the lamin A/C pool in the nuclear interior is lost (Markiewicz et al., 2002, 2005; Naetar et al., 2007). This suggests that LAP2α predominantly functions as a negative cell cycle regulator in proliferating cells, whereas it is not required in post-mitotic cells.

Mutations in *LMNA* cause several human diseases, collectively termed laminopathies (Worman, 2012). One of the most severe laminopathies is the premature aging disease Hutchinson–Gilford progeria syndrome (HGPS) (Gordon et al., 2014; Vidak and Foisner, 2016). This extremely rare genetic disorder reflects many aspects of normal aging, including loss of hair and subcutaneous fat, aged-looking skin, joint stiffness, osteoporosis, atherosclerosis and cardiovascular disease (Gordon et al., 2014). Classical HGPS is caused by a *de novo* heterozygous mutation (1824C>T, p.G608G) in exon 11 of *LMNA* (De Sandre-Giovannoli et al., 2003), which activates a cryptic splice site resulting in the expression of a mutant lamin A, termed progerin (Eriksson et al., 2003). Unlike wild-type (WT) lamin A, progerin remains permanently farnesylated, resulting in its abnormal association with the inner nuclear membrane (Goldman et al., 2004; Reddy and Comai, 2012). Progerin expression induces various cellular defects, including highly lobulated nuclei with thickened lamina, loss of peripheral heterochromatin, compromised DNA repair and chromosome and telomere aberrations, global changes in histone modifications, alterations in several signaling pathways and impaired cell-cycle regulation, resulting in reduced replicative life span and premature senescence (Gordon et al., 2014; Vidak and Foisner, 2016). The exact molecular mechanisms that lead to these cellular defects remain unknown.

We previously reported that LAP2α is downregulated in cultured progerin-expressing cells and that the level of A-type lamins in the nuclear interior is greatly reduced (Vidak et al., 2015). Although loss of LAP2α in proliferating WT cells causes hyperproliferation (Naetar et al., 2008), the reduced levels of LAP2α in cells from HGPS patients (progeria cells) correlate with impaired proliferation. Surprisingly, in contrast to WT cells, overexpression of LAP2α in progeria cells enhances proliferation through upregulation of extracellular matrix (ECM) gene expression (Vidak et al., 2015). These observations led to the hypothesis that LAP2α has a proliferation-inhibiting function in WT cells, probably through its effect on pRb (Dorner et al., 2006; Naetar et al., 2008), and a growth-promoting function in progeria cells, probably by controlling ECM protein expression (Vidak et al., 2015); however, the factors defining whether LAP2α has a growth-promoting or growth-inhibiting function remain unclear. We show here that HGPS patient fibroblasts and progerin-expressing human telomerase reverse transcriptase (hTERT)-immortalized fibroblasts undergo an initial period of hyperproliferation in culture (compared with WT primary human control cells and lamin A-expressing fibroblasts, respectively) before proliferation slows down. In this hyperproliferation state, progerin-expressing cells contain lamin A/C in the nuclear interior and low levels of LAP2α, and respond to LAP2α overexpression by reduced proliferation (similar to WT cells). In later stages in culture, progeria cells that express low LAP2α levels lose nucleoplasmic A-type lamins. Under these conditions, ectopic LAP2α promotes proliferation, whereas simultaneous expression of LAP2α and ectopic lamin A, which rescues the nucleoplasmic pool of lamin A, reduces proliferation. Thus, the proliferation-inhibiting and proliferation-promoting functions of LAP2α in progeria cells depend on the presence and absence of nucleoplasmic lamin A/C, respectively.

## RESULTS

### Progerin-expressing fibroblasts in culture undergo an initial period of hyperproliferation

**Fig. 1.**
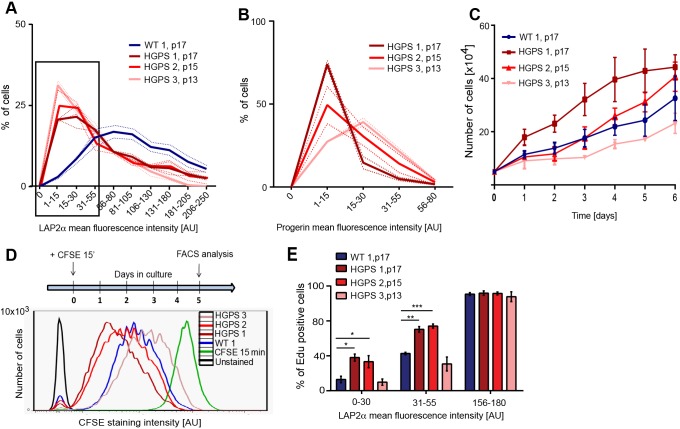
**Progerin-expressing fibroblasts undergo a period of hyperproliferation before going into cell cycle exit.** (A) Mean LAP2α fluorescence intensities of one wild-type (WT 1, p17) and three different HGPS primary human fibroblast cell lines (HGPS 1, p17; HGPS 2, p15; and HGPS 3, p13) were measured in 250 nuclei each and plotted in a histogram (*n*=3). The percentage of nuclei expressing different levels of LAP2α is shown. (B) Histogram of mean progerin fluorescence intensities measured in 300 nuclei each of HGPS cell lines 1, 2 and 3 at indicated passage numbers (*n*=3). Dotted lines represent standard error of mean (s.e.m.). (C) Growth curves of WT 1 and HGPS cell lines 1, 2 and 3 over 6 days (*n*=3). (D) Cells were stained with CFSE for 15 min and grown in complete medium for 5 days before FACS analysis. CFSE staining intensity profiles of WT 1 (blue) and three different HGPS lines (different shades of red) are shown. Green line corresponds to signal after 15 min CFSE pulse and the black profile to the unstained control. (E) WT 1 and three different HGPS lines were grown in medium containing EdU. The percentage of EdU-positive cells was determined after 40 h, followed by immunofluorescence analysis. Mean fluorescence LAP2α intensity of 600 nuclei was measured and plotted against the EdU signal per cell. The percentages of EdU-positive cells in the populations of cells with low LAP2α expression (black rectangle in panel A) can be compared with the percentages in cells with high LAP2α expression. *P<0.05, **P<0.005, ***P<0.0005.

To understand the different effects of LAP2α on proliferation in progeria and WT cells, we analyzed the proliferation properties of mid-passage (p13–p17) dermal fibroblasts derived from HGPS patients and from healthy control individuals in relation to their progerin and LAP2α levels. Data shown here correspond to the HGMDFN168 control cell line (represented as WT 1) and GM04390 cell line (WT 2); control cell lines had similar proliferation phenotypes (see below and Fig. S1D). Three different HGPS cell lines were studied: HGADFN003 (represented as HGPS 1), HGADFN155 (HGPS 2) and AG11513 (HGPS 3). As previously reported (Vidak et al., 2015), LAP2α levels were significantly reduced in HGPS 2 (p15) and HGPS 3 (p13) fibroblasts compared with WT, as demonstrated by immunofluorescence microscopy, whereas HGPS 1 (p17) showed only slight reduction in LAP2α levels, and the extent of LAP2α reduction correlated with progerin expression levels (Fig. S1A–C) (Scaffidi and Misteli, 2005, 2006; Vidak et al., 2015). However, analysis using immunofluorescence microscopy revealed a strong variation in LAP2α expression levels among individual cells in the cultures. To obtain better understanding of potential downstream consequences of LAP2α expression levels in progeria cells, we analyzed these variations systematically. Rather than calculating mean LAP2α fluorescence intensities in the cultures (Fig. S1B), we quantified the LAP2α signal in 250 individual nuclei in WT and HGPS fibroblast cultures and generated LAP2α expression histograms (Fig. 1A). Although the majority of cells in the WT culture expressed high levels of LAP2α, all HGPS cultures showed a notable increase in the percentage of cells expressing low LAP2α levels compared with WT (Fig. 1A, black box), the extent of which correlated with progerin expression levels (Fig. 1B). Furthermore, growth curves of these cultures revealed an interesting proliferation phenotype in progeria versus WT cultures. HGPS lines 1 (p17) and 2 (p15) showed faster proliferation than the control WT line (p17) initially (Fig. 1C), but slower proliferation at later passages (HGPS 2, p21; Fig. S1E). HGPS 3 (p13) displayed slightly reduced proliferation. Importantly, proliferation of three different passage-matched (p17) WT lines (WT 1, WT 2 and HGMDFN090 represented as WT 3) were similar (Fig. S1D). The hyperproliferation phenotype of HGPS lines was confirmed by carboxyfluorescein diacetate succinimidyl ester (CFSE) proliferation assay. The cells were pulse-labeled with CFSE dye for 15 min and chased in complete medium for 5 days before CFSE signals were determined by flow cytometry (Fig. 1D). CFSE is taken up by cells during the pulse and equally segregated into daughter cells as cells divide during the chase period. Thus, the reduction in CFSE intensity correlates with proliferation rates. In agreement with growth curves (Fig. 1C), HGPS lines 1 and 2 showed faster proliferation compared with the control, whereas HGPS 3 showed a slightly slower proliferation (Fig. 1D). Interestingly, whereas HGPS 1 generated a single, uniform CFSE signal, HGPS 2 and 3 cultures presented broader peaks, partially overlapping with the peak of the WT culture (Fig. 1D). The results indicate that these HGPS cultures contain cell populations with different proliferation rates. In addition to a population of cells that proliferate like WT cells, HGPS 2 also contained a faster proliferating population, whereas HGPS 3 contained a population proliferating more slowly than the control. Thus, in addition to the variation in expression levels of LAP2α and progerin (McClintock et al., 2007; Vidak et al., 2015), proliferation is also heterogeneous among cells within HGPS cell cultures.

### Low LAP2α levels in cells with intermediate progerin levels result in hyperproliferation

To investigate a potential correlation between LAP2α expression levels and cell proliferation in HGPS cultures, we measured 5-ethynyl-2′-deoxyuridine (EdU) incorporation over a period of 40 h, followed by immunofluorescence-based analysis of LAP2α protein levels in 600 individual nuclei. Within the population of cells with low LAP2α levels (Fig. 1A, black square), HGPS 1 and HGPS 2 cells showed increased EdU incorporation (i.e. proliferation) compared with WT and HGPS 3 (Fig. 1E). In contrast, no difference in EdU incorporation rates was detectable between WT and HGPS cells expressing high levels of LAP2α (Fig. 1E). Hence, in proliferating HGPS cells expressing intermediate progerin levels (HGPS 1 and 2), some cells might progressively lose LAP2α, which can in turn account for increased proliferation, as previously reported in LAP2α-knockout cells (Naetar et al., 2008). In contrast, cells expressing low levels of LAP2α in WT and HGPS 3 cultures (expressing high progerin levels) showed only background EdU incorporation and could thus represent cell-cycle arrested cells, which have downregulated LAP2α as previously reported in post-mitotic cells (Markiewicz et al., 2002, 2005; Naetar et al., 2007).

**Fig. 2.**
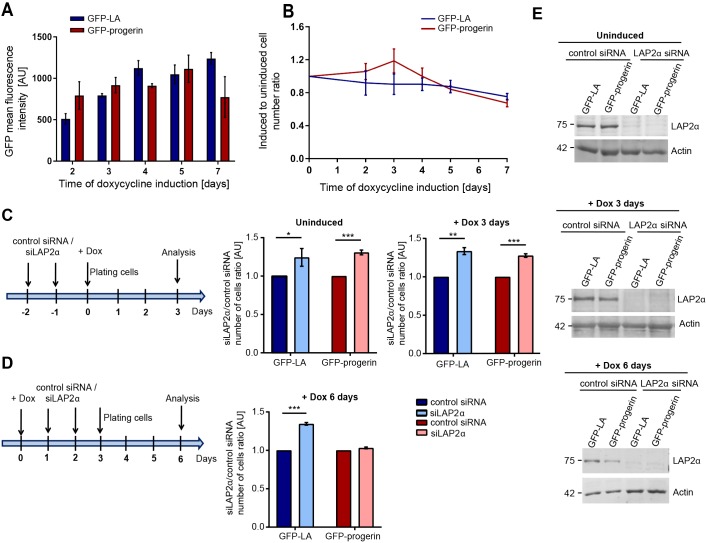
**Loss of LAP2α increases proliferation of WT cells and cells with low progerin expression.** (A) Mean GFP-lamin A (GFPLA) and GFP–progerin fluorescence intensities were measured in 250 nuclei each from hTERT-immortalized fibroblasts following doxycycline induction of GFP–progerin (0.5 µg ml^−1^ doxycycline) and GFP–LA (0.1 µg ml^−1^ doxycycline) for 2, 3, 4, 5 and 7 days, and plotted in a histogram (*n*=3). (B) Growth curves of hTERT-immortalized fibroblasts inducibly expressing GFP–LA or GFP–progerin grown in the presence of 0.1 and 0.5 µg ml^−1^ doxycycline, respectively, for 7 days. The number of cells was normalized to the respective uninduced control (*n*=3). (C) hTERT-fibroblasts inducibly expressing GFP–LA or GFP–progerin were transfected either with a scrambled control siRNA or siRNA targeting LAP2α (siLAP2α) on two consecutive days prior to doxycycline induction. The cell number was determined after 3 days cultivation in the absence (−Dox) or presence (+Dox) of 1 µg ml^−1^ doxycycline (*n*=3). The results are shown as the ratio of cell numbers for siLAP2α versus scrambled siRNA control (*n*=3). (D) GFP–LA and GFP–progerin expression was induced in hTERT-immortalized fibroblasts by addition of 1 µg ml^−1^ doxycycline for 24 h. The cells were transfected either with a scrambled control siRNA or siLAP2α on two consecutive days and the cell number determined at post-induction day 6 (*n*=3). The results are shown as the ratio for siLAP2α versus scrambled siRNA control (*n*=3). (E) Immunoblot analysis of total cell lysates using anti-LAP2α and anti-actin (loading control) antibodies. *P<0.05, **P<0.005, ***P<0.0005.

To investigate a potential causal link between LAP2α expression levels and proliferation in progerin-expressing cells, we used a tightly controllable hTERT-immortalized skin fibroblast system allowing doxycycline-inducible expression of GFP–progerin or GFP–lamin-A as control (Vidak et al., 2015). Progerin and lamin A levels were detected after one day of doxycycline induction and increased within 6–8 days, as revealed by fluorescence intensity measurements and immunoblotting of cell lysates (Fig. 2A; Fig. S2A). Exogenous GFP–progerin levels were similar to endogenous lamin A levels; those of GFP–lamin-A were up to twofold higher than endogenous lamin A levels in the presence of 0.5 µg ml^−1^ doxycycline (Fig. S2A). We then assessed cell proliferation following addition of 0.5 µg ml^−1^ doxycycline for GFP–progerin and 0.1 µg ml^−1^ doxycycline for GFP–lamin-A cells relative to the respective uninduced controls (Fig. 2B). Importantly, at these doxycycline concentrations, expression levels of GFP–lamin-A and GFP–progerin were comparable (Fig. 2A). Proliferation of cells expressing GFP–lamin-A was slightly reduced compared with uninduced cells; cells expressing GFP–progerin initially (1–4 days induction) proliferated faster than uninduced controls and cells expressing GFP–lamin-A. At days 5–8 post-induction, when progerin levels further increased (Fig. S2A), GFP–progerin fibroblasts exhibited slower proliferation. Hence, in agreement with our observation in HGPS cells (see above) and previous reports (Bridger and Kill, 2004), these data suggest that lower progerin levels correlate with increased proliferation compared with controls, whereas higher progerin expression levels eventually lead to reduced proliferation. Together, these data indicate a progerin-specific growth-promoting phenotype at initial phases of induction.

To test whether the initial hyperproliferation of progerin-expressing cells is causally linked to reduced LAP2α levels, as previously proposed (Chojnowski et al., 2015), we analyzed the effect of siRNA-mediated downregulation of LAPα at different stages of doxycycline induction. Human TERT-fibroblasts were transfected with a siSCRAMBLE control oligonucleotide or with siRNA oligonucleotide targeting LAP2α, which caused downregulation of LAP2α protein by ∼90% (Fig. 2E). In uninduced cells and cells expressing GFP–progerin or GFP–lamin-A after 3 days of doxycycline induction, downregulation of LAPα resulted in increased proliferation (Fig. 2C), as previously reported for WT cells (Naetar et al., 2008). Thus, under these conditions (intermediate progerin expression and low levels of LAP2α), progerin-expressing cells behave like WT cells in proliferation assays. However, after 6 days of ectopic protein induction, progerin-expressing cells proliferated more slowly and no longer showed hyperproliferation upon LAP2α downregulation, whereas proliferation of lamin A-expressing control cells remained unchanged (Fig. 2D). Thus, the proliferation response of cells to LAP2α downregulation differs for cells with low or high levels of progerin expression.

### LAP2α-mediated rescue of HGPS proliferation depends on loss of nucleoplasmic lamins

**Fig. 3.**
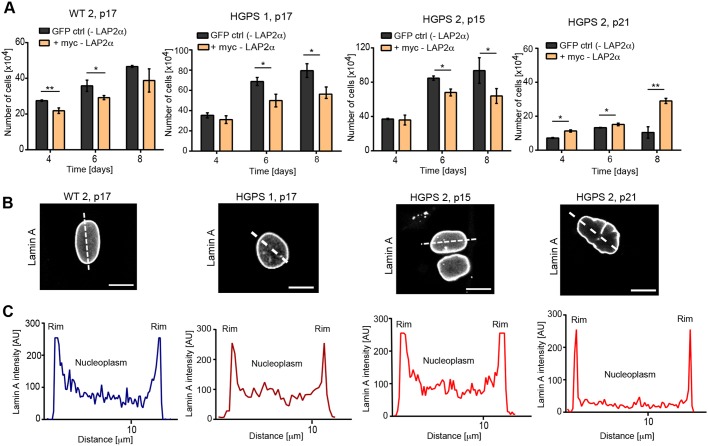
**Effect of ectopic LAP2α expression on proliferation of patient cells depends on the levels of nucleoplasmic lamins.** (A) Human primary fibroblasts were transfected either with a control GFP-expressing construct (GFP ctrl) or a human myc-LAP2α-expressing construct (myc-LAP2α) on two consecutive days and counted on days 4–8 (*n*=3). (B) Primary human fibroblasts were fixed with 4% paraformaldehyde and processed for immunofluorescence using anti-lamin A specific antibody. Scale bars: 10 µm. (C) Fluorescence intensity of the lamin A signal was measured across nuclei (dotted line in B) and plotted. *P<0.05, **P<0.005.

Previous studies (Chojnowski et al., 2015; Vidak et al., 2015) have shown that ectopic expression of LAP2α in HGPS patient cells rescues cell proliferation, whereas it reduces proliferation in WT cells. To test whether this growth-promoting effect of LAP2α in progeria cells also varies with progerin levels, we introduced myc-tagged human LAP2α or GFP as a control into progeria cells derived from young patients (HGPS 1, p17; HGPS 2, p15) expressing intermediate levels of progerin (Fig. S1C), and into HGPS 2 at later passages (p21) expressing higher levels of progerin (Vidak et al., 2015). Cells were transfected on two consecutive days, followed by analysis of expression levels of lamin A/C, progerin and myc–LAP2α by immunoblotting (Fig. S2C), and analysis of cell proliferation. Unlike GFP expression, ectopic expression of LAP2α negatively affected proliferation in WT cells (Fig. 3A), consistent with previous data (Dorner et al., 2006). However, in contrast to previous reports (Chojnowski et al., 2015; Vidak et al., 2015), LAP2α expression also decreased the proliferation of HGPS 1 cells at passage 17 and HGPS 2 cells at passage 15 (Fig. 3A). Only in late passage HGPS 2 cells (p21) did expression of LAPα significantly increase proliferation (Fig. 3A).

To elucidate why LAP2α expression rescues cell proliferation in late passage HGPS cells, but slows down proliferation in mid-passage cells, we tested the level of nucleoplasmic lamin A, previously shown to be downregulated in progeria cells (Vidak et al., 2015). Immunofluorescence microscopy using a lamin A specific antibody (not detecting progerin) revealed that nucleoplasmic lamins are still present in mid-passage progeria cells (HGPS 1 at p17 and HGPS 2 at p15), but absent in late passage HGPS 2 (p21) cells (Fig. 3B,C; Fig. S3). These data suggest that the effect of LAP2α on the proliferation of progerin-expressing cells depends on the level of nucleoplasmic lamins, whereby high nucleoplasmic lamin levels result in decreased proliferation and low nucleoplasmic lamin levels result in a proliferation-promoting effect of ectopic LAP2α.

**Fig. 4.**
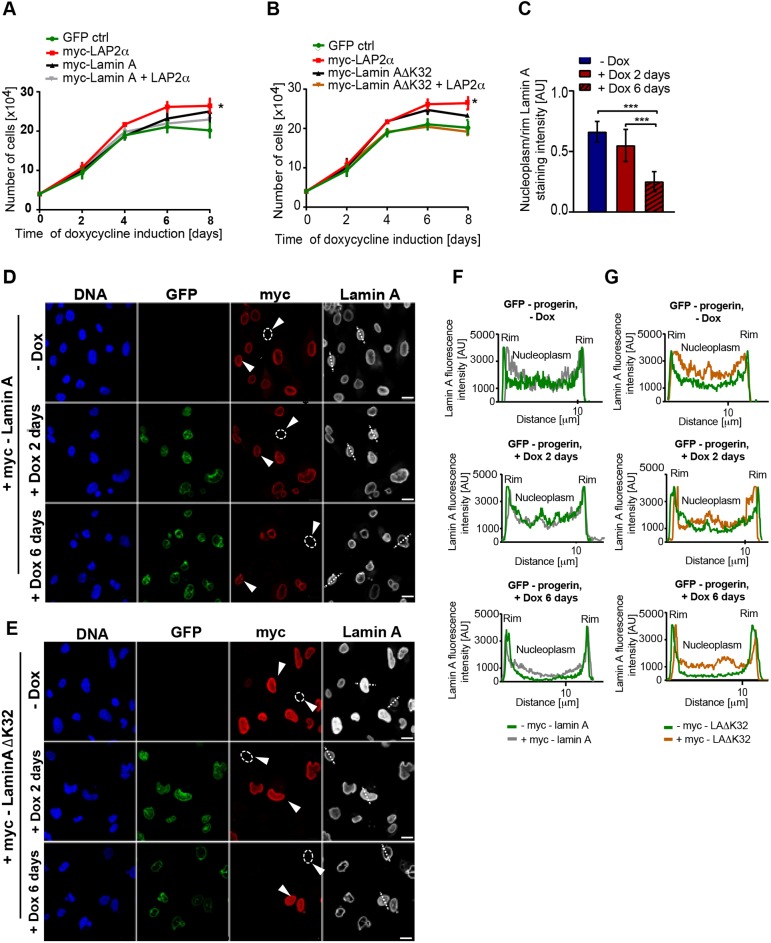
**Re-expression of LAP2α in the presence of nucleoplasmic lamins does not rescue proliferation of progerin-expressing cells.** (A) hTERT-immortalized fibroblasts inducibly expressing GFP–progerin were transfected either with a single construct (control GFP-expressing plasmid, human myc-lamin A- or human myc-LAP2α-expressing construct) or with a combination of myc–lamin A- and myc-LAP2α-expressing constructs on two consecutive days prior to the induction of progerin expression and counted on days 2–8 post-induction (*n*=3). *P<0.05 comparing cells expressing myc-LAP2α with cells expressing myc-lamin A+LAP2α. (B) The same as for A, except that myc–laminAΔK32 expressing construct was used instead of WT lamin A-expressing construct (*n*=3). *P<0.05 comparing cells expressing myc-LAP2α with cells expressing myc–lamin AΔK32+LAP2α. (C) Ratios of nucleoplasmic to peripheral mean lamin A fluorescence intensities were calculated from 100 GFP–progerin-expressing nuclei in immunofluorescence images and plotted in a histogram (***P<0.0005). (D) Immunofluorescence analysis of cells transfected with human myc-lamin A using anti-myc (red) and lamin A-specific antibody (greyscale). DAPI (blue) was used to stain DNA. (E) Immunofluorescence analysis of cells transfected with human myc-lamin AΔK32 using anti-myc (red) and lamin A-specific antibody (greyscale). DAPI (blue) was used to stain DNA. Circles in D and E indicate cells not expressing myc-lamin A or myc-lamin AΔK32 and arrowheads point to the cells represented in the intensity profiles in F and G. (F,G) Mean fluorescence intensities of the lamin A signal were measured across nuclei (dotted lines in D and E) and plotted. Scale bars: 20 µm.

Similar effects were observed in the controllable GFP–progerin-expressing hTERT-fibroblast line. After 6 and 8 days, expression of LAP2α in doxycycline-induced progerin-expressing cells, which have high progerin levels (Fig. S2A) and low levels of nucleoplasmic lamins (Fig. 4C), significantly enhanced cell proliferation to the level of cells expressing GFP–lamin-A (6 days) or by ∼50% compared with the control at 8 days (Fig. 4A,B; Fig. S4A). To rescue the nucleoplasmic lamin A pool, we expressed either myc-tagged WT lamin A or a myc-tagged mutant ΔK32 lamin A. The latter is a lamin variant linked to a severe form of congenital muscular dystrophy (CMD) in humans (Quijano-Roy et al., 2008). In *Lmna^ΔK32/ΔK32^* knock-in mice, ΔK32 lamin A failed to assemble into the lamina and mislocalized to the nucleoplasm (Bertrand et al., 2012; Pilat et al., 2013). Using a lentiviral transduction system, we achieved a transfection efficiency of up to 90% in hTERT-immortalized fibroblasts; myc-tagged proteins were readily detectable by immunoblot analysis (Fig. S4C,D). Immunofluorescence analysis using an anti myc-tag antibody revealed that WT lamin A localized mostly at the nuclear periphery, but a small fraction was also detected in the nuclear interior, whereas the laminAΔK32 mutant was present predominantly in the nuclear interior, significantly enriching the nucleoplasmic lamin pool (Fig. S4B). For cell proliferation analyses, cells expressing GFP–progerin were transduced with control constructs encoding WT lamin A, laminAΔK32, LAP2α or GFP, and with a combination of constructs encoding lamin A plus LAP2α or laminAΔK32 plus LAP2α on two consecutive days prior to the induction of GFP–progerin. Analyses were carried out up to 8 days post-induction (Fig. 4A,B). The nucleoplasmic pool of lamin A was partially restored by ectopic lamin A expression (Fig. 4D,F, arrowheads) and significantly enriched upon expression of laminAΔK32 (Fig. 4E,G, arrowheads). Fibroblasts transfected with a GFP control plasmid showed slower proliferation 4–6 days after progerin induction (Fig. 4A,B), whereas ectopic expression of LAP2α enhanced proliferation of the progerin-expressing hTERT-fibroblasts (Fig. 4A,B). This increase in cell proliferation correlated with loss of nucleoplasmic lamins, as observed by measuring the ratio of nucleoplasmic lamin A staining to rim staining in 100 cells (Fig. 4C). Interestingly, ectopic expression of either WT lamin A or laminAΔK32 mutant alone also led to an increase in proliferation compared with progerin-expressing control cells (Fig. 4A,B). Strikingly, however, ectopic expression of LAP2α together with either WT lamin A or laminAΔK32 (rescuing the nucleoplasmic lamin A pool) almost completely inhibited the proliferation-promoting effect of LAP2α (Fig. 4A,B). Together, our data demonstrate that the effect of LAP2α expression on proliferation in progeria cells is highly dependent on the level of nucleoplasmic A-type lamins.

## DISCUSSION

In this study, we analyzed the proliferation phenotype in progerin-expressing and WT cells in the absence and presence of LAP2α and its relation to the levels of progerin and nucleoplasmic A-type lamins. The HGPS cell lines tested not only showed highly heterogeneous expression levels of LAP2α and progerin within cells in the culture, as previously reported (Vidak et al., 2015), but they also showed heterogeneous proliferation rates. HGPS cell lines expressing intermediate levels of progerin proliferated faster than the respective control cell line, potentially linked to a cell population in the culture with low LAP2α levels. In contrast, HGPS cell cultures expressing high levels of progerin exhibited slower proliferation. Because this variability in different HGPS cell lines could be partially rooted in the different genetic background of patients from whom the cell lines were derived, we also analyzed hTERT-expressing fibroblasts that can be cultured under tightly controllable conditions. Using this cell system, we observed a similar initial hyperproliferation following induction of progerin expression compared with lamin A expression, and a subsequent rapid decline in the number of proliferative cells at later stages, when progerin levels were high.

Intriguingly, a significant fraction of HGPS cells with low LAP2α expression levels still proliferated, indicating that the loss of LAP2α in HGPS cells does not immediately lead to proliferation defects as previously reported (Chojnowski et al., 2015; Vidak et al., 2015). Indeed, these proliferating and LAP2α-negative cells in HGPS cultures could account for the overall hyperproliferation of the cultures, based on previous observations showing that loss of LAP2α in WT fibroblasts increases proliferation (Dorner et al., 2006; Naetar et al., 2008). Thus, LAP2α might have a dual role in the development of HGPS, depending on progerin levels and disease progression. In cells with low progerin levels, reduced levels of LAP2α could cause an initial period of hyperproliferation, whereas at later stages, when progerin expression increases, low LAP2α levels correlate with decreased proliferation. Accordingly, overexpression of LAP2α in cells expressing low progerin levels impairs proliferation, whereas it rescues proliferation in cells expressing high progerin levels. However, how LAP2α is downregulated in a subset of proliferating cells in growing HGPS cultures remains elusive.

Why does loss or gain of LAP2α have different effects in cells with low or high expression of progerin? Immunofluorescence analysis revealed that low progerin-expressing cells, such as progeria lines derived from young patients, contained significant levels of lamin A in the nuclear interior, whereas the nucleoplasmic pool of A-type lamins was greatly reduced in progeria cells derived from an older patient that express higher levels of progerin. In addition, prolonged cultivation of cells derived from young patients, which is known to increase progerin levels (Vidak et al., 2015), significantly decreases the nucleoplasmic pool of lamins. Is it indeed the presence or absence of lamin A/C in the nuclear interior that defines whether LAP2α has proliferation-promoting or proliferation-inhibiting functions? To address this question, we expressed LAP2α alone or in combination with ectopic lamin A in cells expressing GFP–progerin that had lost lamin A/C in the nuclear interior. Expression of LAP2α, which does not rescue the nucleoplasmic pool of lamin A/C in these cells, increased cell proliferation (Vidak et al., 2015). In contrast, ectopic expression of LAP2α together with ectopic WT lamin A or an assembly-deficient disease-linked laminAΔK32 mutant rescued the nucleoplasmic pool of lamin A to different extents but, importantly, did not enhance cell proliferation. Thus, LAP2α primarily has a growth-inhibiting effect in cells containing lamins in the nuclear interior, but a growth-promoting effect in cells that lack nucleoplasmic lamins.

How are these different functions of LAP2α mediated? LAP2α and A-type lamins were found to interact with the cell cycle regulatory protein pRb (Markiewicz et al., 2002), which represses E2F-dependent transcription to mediate cell cycle arrest (Kaelin, 1999). In addition, LAP2α interacts with E2F/pRb target genes and represses an E2F-dependent reporter gene dependent on the presence of pRb (Dorner et al., 2006). The observation that the expression of WT LAP2α in *Lap2α^−/−^* cells, but not the expression of a LAP2α truncation mutant lacking its pRb and lamin A interaction domains, reduced cell proliferation (Naetar et al., 2008) suggests that LAP2α can inhibit proliferation only in collaboration with or in a complex with nucleoplasmic lamin A and pRb (Dorner et al., 2006; Naetar et al., 2008; Pilat et al., 2013). Thus, LAP2α has a proliferation-inhibiting effect only in cells containing lamin A/C in the nuclear interior. This effect is probably mediated at the G1–S phase transition of the cell cycle (Dorner et al., 2006).

Upon progerin accumulation in prolonged cell culture, the nucleoplasmic pool of A-type lamins is lost, probably by forming heteromeric complexes with progerin at the nuclear periphery. This renders LAP2α no longer capable of inhibiting cell proliferation via pRb and its proliferation-promoting function becomes evident. Potential mechanistic insight into its growth-promoting effect are provided by our recent finding that LAP2α interacts with euchromatic regions in the mammalian genome and regulates lamin-A–chromatin association (Gesson et al., 2016). Contrary to previous reports showing preferential binding of lamin A to heterochromatic regions termed lamina-associated domains (LADs) (Kind et al., 2015), we showed that lamin A also binds to euchromatic regions outside of LADs largely overlapping with LAP2α-associated genomic regions in the nucleoplasm. Loss of LAP2α caused a reorganization of lamin A–chromatin association, leading to changes in epigenetic profiles and gene expression (Gesson et al., 2016). Thus, loss of nucleoplasmic lamin A in progeria cells can change epigenetic pathways and gene expression. In line with this hypothesis, we found decreased expression of extracellular matrix genes in progeria versus WT cells and rescue of ECM gene expression upon ectopic expression of LAP2α (Vidak et al., 2015). Although the ECM has previously been shown to affect cell proliferation of progeria cells (de la Rosa et al., 2013; Hernandez et al., 2010; Vidak et al., 2015), the molecular pathways are not completely understood, particularly regarding if and which cell cycle phases are affected.

Interestingly, the *LMNA* cryptic splice site mutated in HGPS is also sporadically used in cells and tissues of elderly apparently healthy individuals, resulting in the production of progerin (McClintock et al., 2007; Scaffidi and Misteli, 2006). LAP2α is downregulated in cells derived from older donors (Miller et al., 2013; Scaffidi and Misteli, 2006), suggesting that progerin and LAP2α could have an important role in normal human ageing.

Together, our studies show that LAP2α has at least two different functions, a growth-inhibiting activity requiring the presence of lamin A/C in the nuclear interior and a growth-promoting role in the absence of nucleoplasmic lamin A/C.

## MATERIALS AND METHODS

### Cell culture

The hTERT-Teton-Pro cell lines were generated and maintained in medium containing 15% Tet-free FBS (Clontech, #631106) as described (Vidak et al., 2015). Protein expression was induced by doxycycline hydrochloride (Sigma, #D9891) at concentrations of 0.1 and 1 μg ml^−1^ for GFP–lamin-A cells and 0.5 and 1 μg ml^−1^ for GFP–progerin cells. Primary dermal fibroblast cell lines from progeria patients and healthy donors were obtained from the Progeria Research Foundation (PRF, Peabody, MA) and the Coriell Cell Repository (CCR, Camden, NJ). HGPS cell lines used were AG11513 (12 years, CCR) at passage 13–16, HGADFN155 (1 year, PRF), at passage 15–21 and HGADFN003 (2 years, PRF) at passage 17. Control cell lines were GM04390 (23 years, CCR), HGMDFN168 (40 years, PRF) and HGMDFN090 (37 years, PRF) used at passage 17. Primary fibroblasts were cultured as described (Vidak et al., 2015). HEK293T cells were cultivated in Dulbecco's modified Eagle's medium (DMEM) containing 10% FBS, 2 mM l-glutamine, 100 U ml^−1^ penicillin and 100 μg ml^−1^ streptomycin.

### Plasmid cloning and lentiviral infections

pHR′ CMV GFP lentiviral plasmid [Addgene, plasmid #14858, deposited by Inder Verma (Miyoshi et al., 1997); a gift from Dieter Blaas, MFPL] was used to generate human myc-LAP2α-pHR lentiviral construct as described (Vidak et al., 2015). For generation of human myc-lamin A-pHR and human myc-lamin AΔK32-pHR lentiviral constructs, pHR′ CMV mCherry plasmid (Ivan Yudushkin, MFPL, Medical University Vienna, Vienna, Austria) was modified as follows: mCherry cassette was deleted with *Not*I and *Bam*HI restriction enzymes and the *Spe*I site was introduced by insertion of oligonucleotides (5′-GATCCCGACTAGTCGGC-3′ and 5′-GGCCGCCGACTAGTCGG-3′), creating pHR′ CMV *Spe*I. cDNA encoding human myc-lamin-A was amplified from pEGFP-C1-lamin A vector with internal myc-tag (provided by Stephen A. Adam, Northwestern University Feinberg School of Medicine, Chicago, IL, USA) (Moir et.al., 2000) and the cDNA encoding human myc-lamin AΔK32 from pEGFP-C1-lamin AΔK32 (Christian Knapp, MFPL, Medical University Vienna, Vienna, Austria) by PCR using 5′-TGTACTAGTGCCATGGAGCAAAAGC-3′ and 5′-TGTACTAGTGAGATCTAATGTACTAC-3′ primers and cloned into pHR′CMV*Spe*I using *Spe*I, generating human myc-lamin A-pHR and human myc-lamin AΔK32 plasmids.

For lentiviral production, HEK293T cells grown on a 10 cm plate were co-transfected with psPAX2 packaging plasmid (5 µg) and pMD2.G envelope plasmid (1.7 µg) (Addgene, plasmid #12259, deposited by Didier Trono) together with myc-LAP2α-pHR, myc-lamin A-pHR, myc-lamin AΔK32-pHR or GFP-expressing lentiviral vector (3.3 µg) using polyethylenimine (PEI; Polysciences). A 30 µl aliquot of PEI stock solution (1 mg ml^−1^ PBS, pH 4.5) was mixed with 10 µg plasmid DNA in 300 µl of OptiMEM and added dropwise to medium on cells. Cells were maintained in 10 ml DMEM, 10% FBS and 2 mM l-glutamine. Viral supernatants were collected after 48 h and 72 h and filtered through 0.45 µm filter. hTERT-TetOn-Pro cells and primary human fibroblasts were plated 24 h prior to infection at a density of 1.5×10^5^ cells per well in a six-well plate. After addition of the 48 h viral supernatant, cells were spun at 1000 ***g*** for 90 min. After 24 h incubation at 37°C, the viral supernatant was replaced with fresh viral supernatant (72 h) and incubated for 24 h.

### siRNA-mediated knockdown of LAP2α

hTERT-TetOn-Pro cells were plated at 70–80% confluence in high glucose DMEM, 15% FCS and 0.2 mM l-glutamine 16 h prior to transfection. For a 10 cm^2^ culture well (six-well) 5 µmol siRNA and 5 μl DharmaFECT (Thermo Scientific, #T-2001-01) were used according to the manufacturer's instructions. Human LAP2α-specific siRNAs (targeted region cDNA 1228–1250) used for transfection were sense siRNA 5′-GAGAAUUGAUCAGUCUAAGdTdT-3′ and an antisense siRNA 5′-CUUAGACUGAUCAAUUCUCdTdT-3′ (Sigma). Silencer negative control siRNAs (siSCRAMBLED; unspecific) were sense siRNA 5′-GAAUCUGACUAGUUAAGAGdTdT-3′ and antisense siRNA 5′-CUCUUAACUAGUCAGAUUCdTdT-3′ (Sigma).

### Proliferation assays

For growth curves, hTERT-TetOn-Pro cell lines or primary human fibroblasts were plated in triplicates on six-well plates at a density of 40,000 cells per well and grown for 7 days under the indicated conditions (0.1–1.0 µg ml^−1^). For the siRNA experiments, cells were plated at a density of 100,000 cells per well and grown for 3 days. Cell numbers were determined using a Casy cell counter model TTC (Schärfe System). EdU incorporation assays were performed using the EdU–Click imaging kit according to the manufacturer's manual (Base Click). Positive cells were quantified using a LSM 710 confocal microscope (Carl Zeiss) and 25×0.8 NA oil immersion objective.

The CFSE staining assay was performed using Vybrant^®^ CFDA SE Cell Tracer Kit (Molecular Probes, V12883). A stock solution of 10 mM CFDA SE (carboxyfluorescein diacetate succinimidyl ester; CFSE) was prepared freshly by dissolving the contents of one vial (component A) in 90 µl high-quality DMSO (component B) and diluting in PBS to 20 µM. Cultures (at 40% confluence) of primary human fibroblasts on a 6 cm plate were incubated with 20 µM CFSE probe in pre-warmed (37°C) PBS for 15 min at 37°C. Cultures were grown in fresh complete medium for an additional 5 days at 37°C, washed once with PBS, trypsinized, collected in a 15 ml Falcon tube and pelleted by centrifugation at 1100 rpm for 5 min. The cell pellet was resuspended in 1 ml of PBS and the CFSE signal detected by flow cytometry using the BD FACS Calibur multicolor flow cytometer (BD Biosciences).

### Immunofluorescence and immunoblotting

Cells grown on glass coverslips were fixed for 15 min with 4% paraformaldehyde at room temperature and incubated with primary and secondary antibodies (DyLight Fluor Secondary Antibodies, Thermo Scientific) as described (Vidak et al., 2015). Samples were counterstained with DAPI (1:15,000 in PBS) for 15 min and mounted in glycerol mounting medium (Antifade 2, DABCO). Images were acquired on a LSM 700 confocal microscope (Carl Zeiss) using 40×1.3 NA oil immersion objective Plan-Apochromat or 63×1.4 NA oil differential interference contrast Plan-Apochromat (Carl Zeiss). Images were exported using ZEN software (Carl Zeiss) and processed using Adobe Photoshop CS3. Tile scan images used for mean fluorescence intensity measurements were acquired on a LSM 710 confocal microscope (Carl Zeiss) using 25×0.8 NA oil immersion objective, 1× zoom and 5×5 tile scan. Mean fluorescence intensities were quantified using ImageJ. The intensity of nucleoplasm and rim staining was measured using the profile tool in ZEN image software (Carl Zeiss). Peripheral and nucleoplasmic intensity values were determined along a nuclear axis and plotted.

For immunoblotting, total cell lysates from 7.5×10^5^ cells in 150 µl of 1× SDS sample buffer (186 mM Tris-HCl, pH 6.8, 30% glycerol, 6% SDS, 300 mM DTT, 0.1% Bromophenol Blue) were processed as described (Vidak et al., 2015) using horseradish peroxidase-labeled secondary antibodies. Quantification of protein levels was performed using the ChemiDoc system (Bio-Rad).

Primary antibodies used for immunofluorescence and immunoblotting were rabbit antiserum to LAP2α (clone 245.2, 1:1000) (Vlcek et al., 2002); mouse monoclonal anti-LAP2α (clone 15/2, undiluted hybridoma supernatant) (Vlcek et al., 2002); rabbit polyclonal anti-lamin A (1:1000; Dechat et al., 2007, provided by Robert Goldman, Northwestern University Feinberg School of Medicine, Chicago, IL, USA); mouse monoclonal anti-progerin, clone 13A4 (1:50, cat. no. 39965, Active Motif, provided by Egon Ogris, MFPL, Medical University Vienna, Vienna, Austria); monoclonal mouse anti-myc (1:1000) provided by Egon Ogris (clone 4A6, cat. no. 05-724, Merck-Millipore); mouse monoclonal anti-lamin A/C antibody (1:5000, clone E1, cat. no. sc-376248, Santa Cruz Biotechnology); mouse monoclonal anti-γ-tubulin (1:5000, clone GTU-88, cat. no. T6557, Sigma); and goat polyclonal anti-actin (clone I-19, cat. no. sc-1616, Santa Cruz Biotechnology).

### Statistical analysis

All calculations were performed using Microsoft Excel and Graphpad Prism software. Experimental data are reported as means of a minimum of three biological replicates. The two-tailed Student's *t*-test was used for statistical analyses. Error bars represent standard deviation (s.d.), except in the growth curves where error bars represent the standard error of the mean (s.e.m.). Statistical significance was classified as follows: *P<0.05, **P<0.005 and ***P<0.0005.

## Supplementary Material

Supplementary information
