# Supplementary material for: Nucleoplasmic lamins define growth-regulating functions of lamina-associated polypeptide 2α in progeria cells
Source: J Cell Sci. 2018 Feb 1;131(3):jcs208462. doi: 10.1242/jcs.208462 (PMC5826045; doi:10.1242/jcs.208462)
Supplement: Supplementary information [file joces-131-208462-s1.pdf]

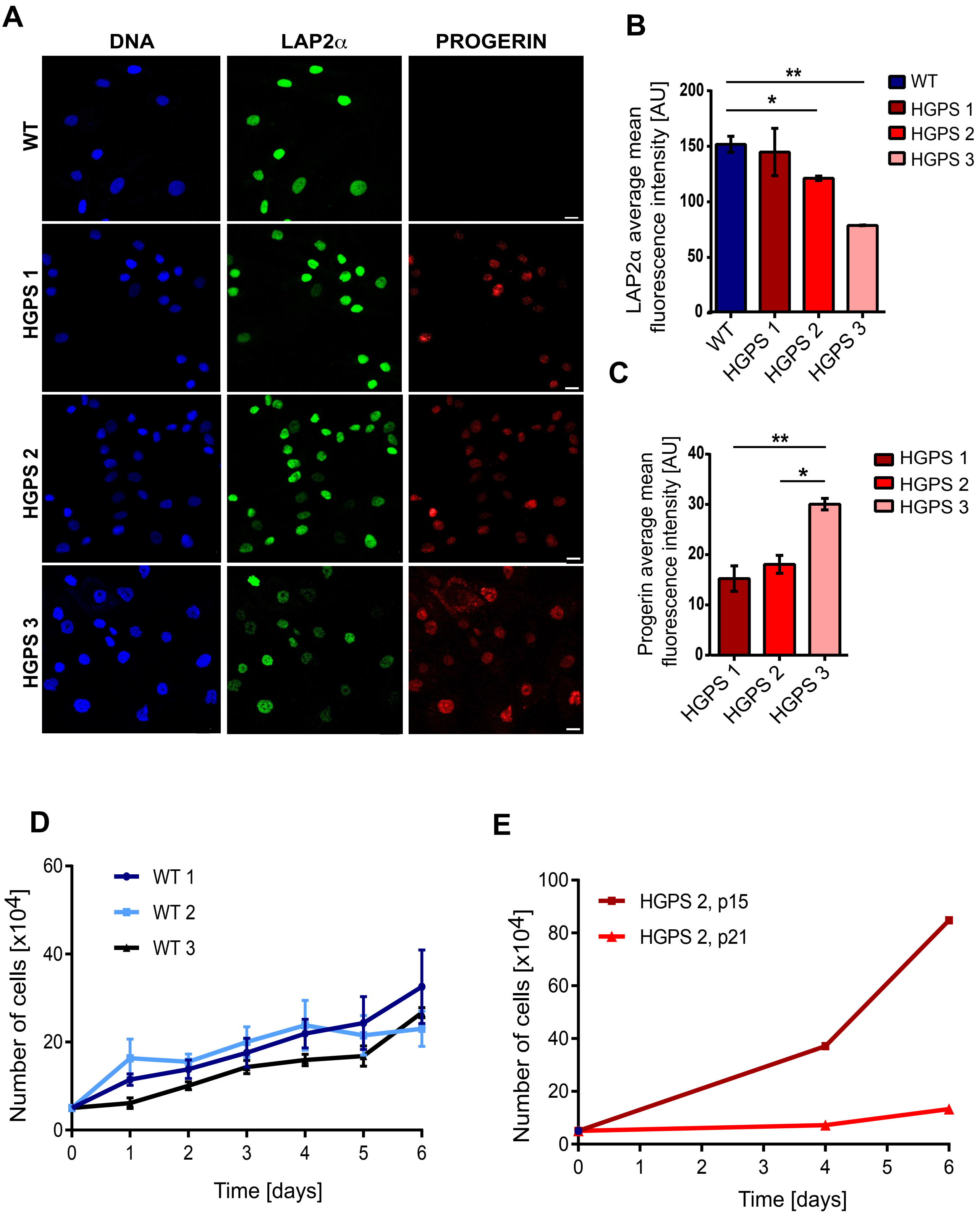

Journal of Cell Science • Supplementary information

Figure S1. LAP2α is downregulated in HGPS fibroblasts. (A) Primary human fibroblasts were fixed with 4% PFA and processed for immunofluorescence using anti LAP2α antibody (green) and progerin antibody (red). DNA was stained with DAPI (blue). Scale bar: 20µm. **(B)** Average mean LAP2α fluorescence intensities of one wild-type (WT 1) and three different HGPS primary human fibroblast cell lines (HGPS 1, 2 and 3) were measured in 250 nuclei each and plotted in a histogram (n=3). **(C)** One wild-type (WT 1) and three different HGPS primary human fibroblast cell lines (HGPS 1, 2 and 3) were stained with anti-progerin antibody. Average mean progerin fluorescence intensities were measured in 250 nuclei each and plotted in a histogram (n=3). **(D)** Growth curves of a three different WT cell lines (p17) over 6 days (n=3). **(E)** Growth curves of HGPS 2 cell line at middle (p15) and late (p21) passage (n=3).

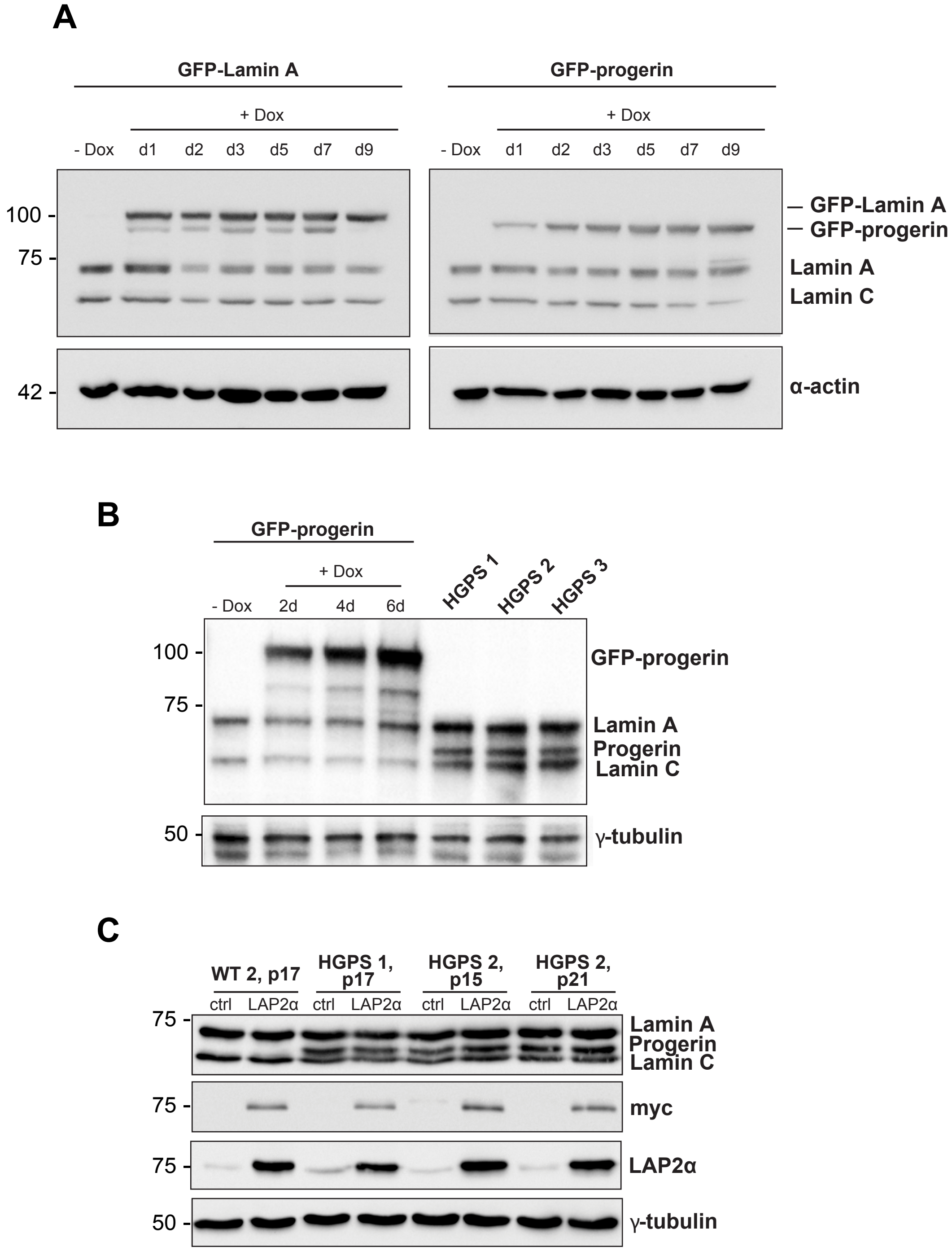

Journal of Cell Science • Supplementary information

Figure S2. Immunoblot analysis of hTERT TetOn cells and primary human fibroblasts. (A) Immunoblot analysis of total cell lysates of GFP-Lamin A and GFP-progerin expressing cells at different time-points after induction with 0.5 μg ml^{-1} doxycycline using anti-lamin A/C antibody. α-ctin levels served as a loading control. **(B)** Immunoblot analysis of GFP-progerin cells at different time-points of induction with 1.0 μg ml^{-1} doxycycline and primary HGPS-derived fibroblasts using anti-lamin A/C antibody. γ-tubulin served as a loading control. **(C)** Immunoblot analysis of one wild-type and three different HGPS cell lines using anti-lamin A/C, anti-myc and anti-LAP2α antibodies following lentiviral transfections with myc-tagged human LAP2α or GFP (ctrl) plasmids. γ-tubulin levels served as a loading control.

Journal of Cell Science • Supplementary information

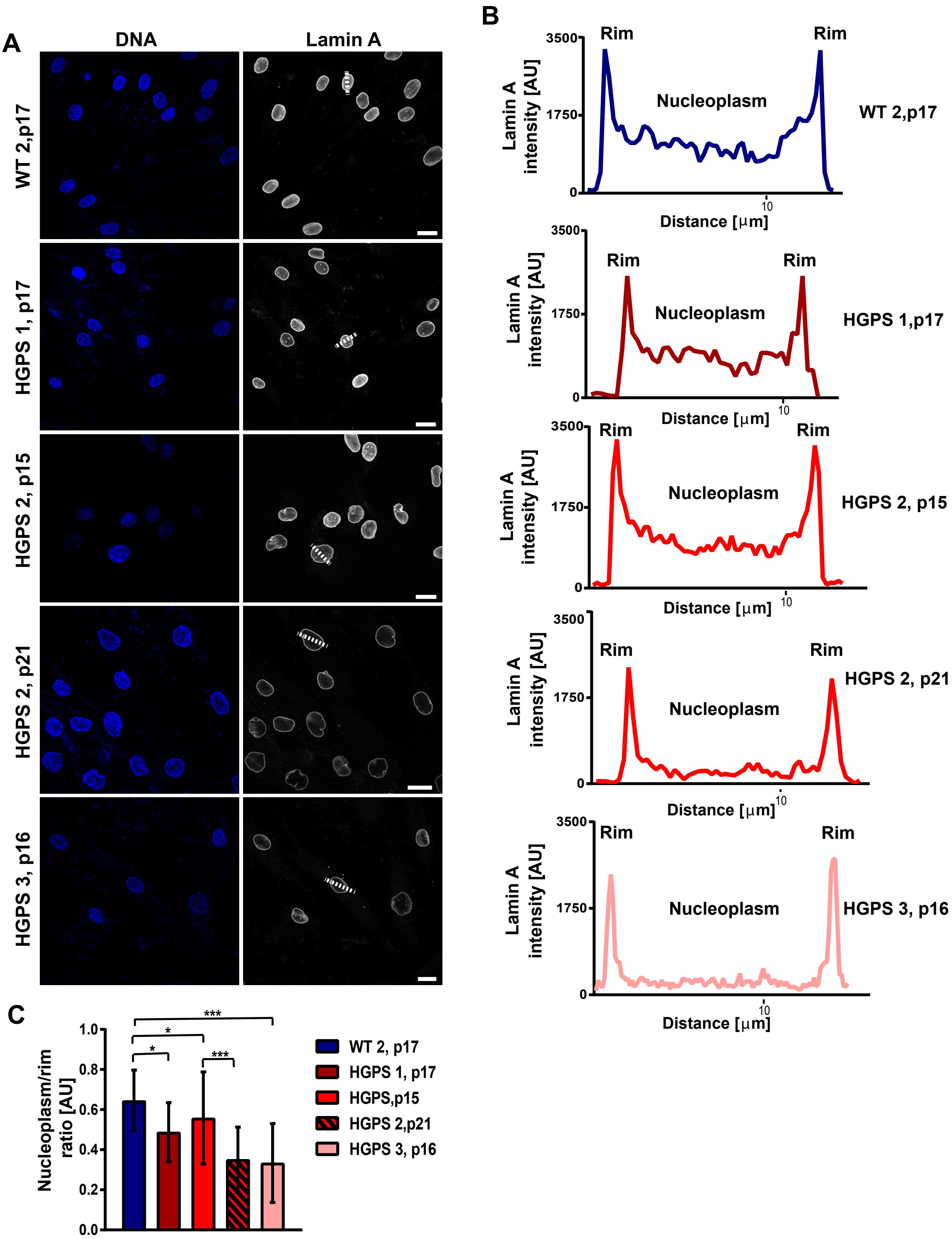
A
DNA
Lamin A
WT 2,p17
HGPS 1, p17
HGPS 2, p15
HGPS 2, p21
HGPS 3, p16
B
Rim
Nucleoplasm
Lamin A intensity [AU]
Distance [µm]
3500
1750
0
10
WT 2,p17
HGPS 1,p17
HGPS 2, p15
HGPS 2, p21
HGPS 3, p16
C
Nucleoplasm/rim ratio [AU]
1.0
0.8
0.6
0.4
0.2
0.0
*
***
WT 2, p17
HGPS 1, p17
HGPS,p15
HGPS 2,p21
HGPS 3, p16

Figure S3. Nucleoplasmic pool of A-type lamins is not affected in low progerin-expressing cells. (A) Primary human fibroblasts were fixed with 4% PFA and processed for immunofluorescence using antibody specifically recognizing lamin A, not lamin C or progerin. DNA was stained with DAPI (blue). Scale bar: 20 µm. **(B)** Fluorescence intensity of the lamin A signal was measured across nuclei (dotted line) and plotted. **(C)** Nucleoplasmic over rim ratio of the lamin A staining was measured in 100 nuclei each of one wild-type and three different HGPS cell lines and plotted in a histogram.

Journal of Cell Science • Supplementary information

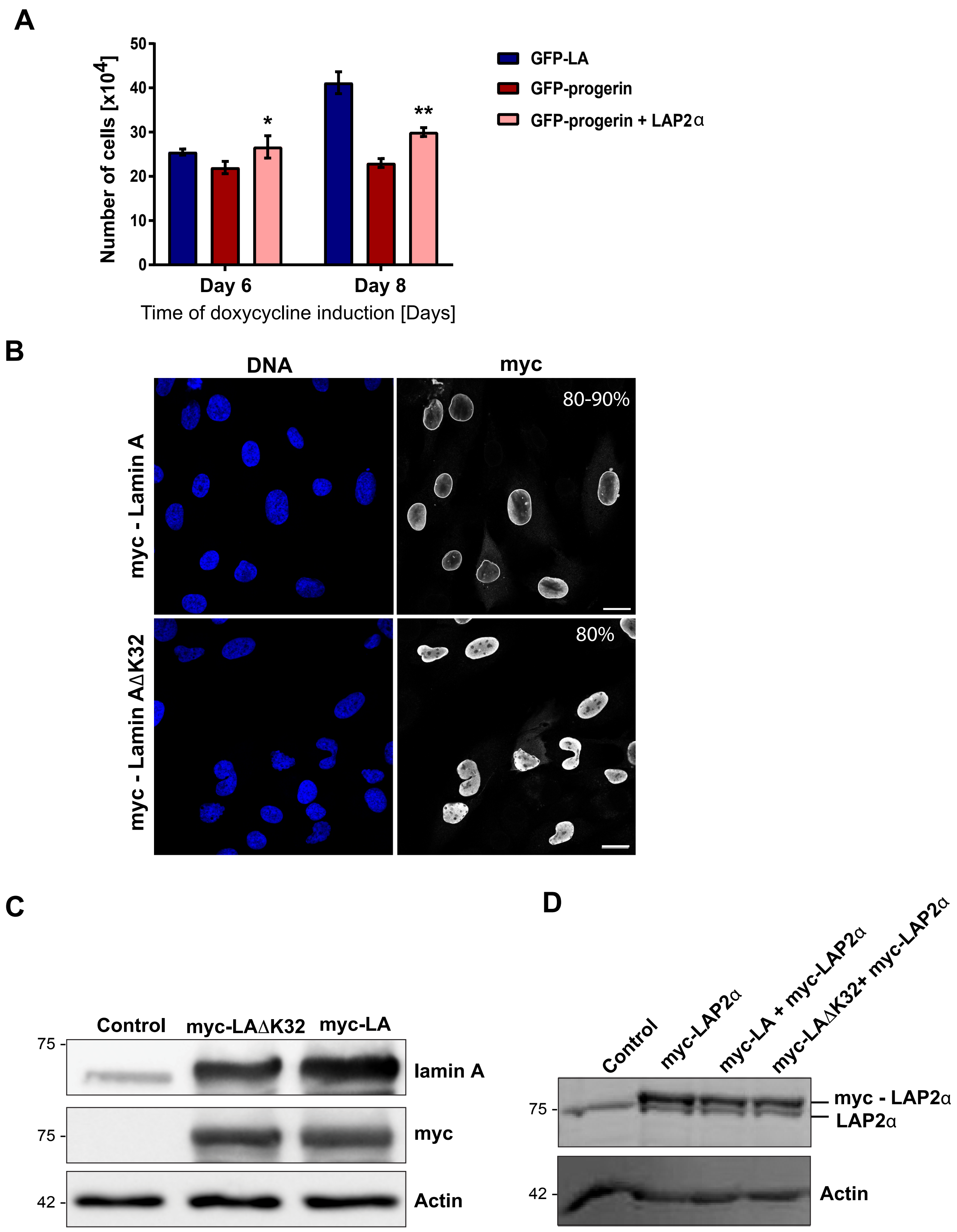

Journal of Cell Science • Supplementary information

Figure S4. Ectopic expression of human myc-Lamin A, human myc-LaminΔK32 and human myc-LAP2α in hTERT-immortalized skin fibroblasts. (A) hTERT–immortalized fibroblasts inducibly expressing GFP-progerin were transfected either with a GFP-expressing (ctrl) or myc-LAP2α expressing construct on two consecutive days prior to the induction of progerin expression and the cell number was determined on days 6 and 8 post-induction (1.0 µg ml^{-1} doxycycline, n=3). GFP-Lamin A expressing cells were used as control. **(B)** Immuno-fluorescence analysis of myc-Lamin A- (upper panel) and myc-LaminAΔK32- (lower panel) expression using α-myc antibody (grey) and DAPI (blue). Scale bar: 20 µm. The percentage indicates transfection efficiency. **(C)** Immunoblot analysis of total cell lysates of myc-Lamin A- and myc-LaminΔK32-expressing cells using anti-myc and anti-lamin A/C antibodies. γ-tubulin levels served as a loading control. **(D)** Uninduced cells were transfected either with a control GFP-expressing construct (GFP ctrl), myc-LAP2α- expressing construct alone or in combination with the myc-Lamin A and myc-LaminAΔK32 construct. The level of LAP2α expression was assessed by immunoblot analysis using anti-LAP2α specific antibody.

Journal of Cell Science • Supplementary information
